# The metal-uptake-deficient Escherichia coli strain GR536 contains the ϕ80 prophage

**DOI:** 10.1099/acmi.0.001204.v3

**Published:** 2026-08-07

**Authors:** Ayuki Shimpo, Jerry Augustine, Luke J. Acton, Lindsay J. Hall, Peter Chivers

**Affiliations:** 1 Department of Biosciences, Durham University, Durham DH1 3LE, UK; 2 Institute of Microbiology and Infection, University of Birmingham, Birmingham, B15 2TT, UK; 3 Department of Microbes, Infection and Microbiomes, School of Infection, Inflammation and Immunology, College of Medicine and Health, University of Birmingham, Birmingham B15 2TT, UK

**Keywords:** ferric, ferrous, import, iron, metal homeostasis, pKD4

## Abstract

We report the genome sequence of *Escherichia coli* GR536, a previously constructed metal-uptake-deficient strain derived from *E. coli* W3110. Growth of GR536 in an iron-restricted liquid medium resulted in apparent lysis during the early exponential growth phase. This effect was exacerbated in cells transformed with pBAD30, a commonly used arabinose-inducible expression vector. Whole-genome sequencing confirmed the expected gene disruptions (*entC*, *feoABC*, *mntH*, *zupT::cat* and *fecABCDE::kan*). However, comparison to *E. coli* W3110 identified the presence of the ϕ80 prophage (46.16 kbp) and cryptic prophage CPZ-55 (6.763 kbp), as well as the absence of cryptic prophage e14 (15.193 kbp). We also identified 9 IS-element deletions, 3 IS-element insertions, 7 other deletions or insertions and 74 candidate individual nucleotide changes. The growth defect in GR536 correlated with lysis due to the production of ϕ80 virions as determined by the inability of isolated phage to infect an *E. coli* strain lacking the phage receptor (∆*fhuA*) and the *Bam*HI digestion pattern of the purified phage DNA. We further determined that the ϕ80-dependent lysis in GR536 is exacerbated by the presence of the chloramphenicol- and kanamycin-resistance markers introduced during construction of GR536 and the pBAD30 plasmid multiple cloning site. Removal of the markers (*E. coli* GR536*) and disruption of the pBAD30 multiple cloning site generated a strain that showed a 10^4^-fold reduction in ϕ80 production. Furthermore, construction of a W3110 lysogen containing the ϕ80 prophage and comparison with GR536* grown under the same conditions showed a ~10^4^-fold higher level of phage production by the parent strain, indicating that phage-dependent lysis was not increased by the deletion of the metal-uptake genes and thus independent of iron availability. These observations clarify growth conditions that limit the effects of ϕ80-dependent lysis when using GR536 to identify metal-uptake genes by complementation, specifically, removal of the antibiotic resistance markers and the avoidance of using intact pBAD30 as a negative control.

## Data Summary

The genome sequence of *Escherichia coli* GR536 has been deposited with GenBank with accession number JBWJAO01000000.1 (BioProject PRJNA1419173). The accession number for the previously published genome sequence of *E. coli* W3110 is AP009048.1 (BioProject PRJNA16351).

## Introduction

Iron uptake by bacteria involves numerous genes that encode multi-protein importers capable of recognizing different Fe-complexes and/or oxidation states [1, 2]. Disruption or deletion of one or more of these systems can provide insight into the identity of the imported iron species and potentially growth conditions under which these transporters are required. *E. coli* GR536 was constructed for this purpose and lacks multiple genes with functions linked to metal uptake (*entC*, *fecABCDE*, *mntH*, *zupT* and *feoABC*) [3]. The strain has since been used to test for complementation by plasmid-borne predicted Fe-uptake systems from different *E. coli* strains [4], other bacterial species [5, 6], or to test metal co-factoring in *E. coli* enzymes [7, 8].

GR536 was constructed [3] prior to the availability of the Keio collection of single knockouts [9], using successive rounds of sequential individual gene inactivation by recombination and removal of selectable markers [10]. P1 transduction was also listed as an experimental method, but it is not clear at what stage, or even if, it was used for GR536 construction. Before using GR536 for complementation experiments similar to those previously reported, we tested growth of GR536 containing a control plasmid, pBAD30, in an iron-restricted growth medium and observed apparent lysis of the cultures. This behaviour had not been noted in past studies, so we carried out whole-genome sequencing of the strain to ensure that its cumulative metal-uptake deficiencies had not resulted in suppressor mutations or other genetic changes. Notably, after whole-genome sequencing, we identified the presence of the ϕ80 prophage. This phage is notable for its less obvious transmission between cells than λ phage [11, 12]. Further characterization of growth behaviour confirmed induction of the ϕ80 prophage in early exponential growth and the production of virions as the likely cause of the lysis. Phage production was observed in all *E. coli* lysogens used in this study under all conditions tested even if lysis was not obvious from changes in optical density, consistent with the previous reports of ϕ80 ‘stealth’ behaviour [11–13], and may indicate why its presence in *E. coli* GR536 had not been previously noted.

## Methods

### Bacterial plasmids, strains and growth

Plasmids pBAD30 [14], pUC18 [15], pMPM-A5 [16] and pMncA [17] were from lab stocks. *E. coli* GR536 was obtained by the Hall laboratory from the Nies lab [3]. *E. coli* W3110 and BW25113 were from lab stocks. *E. coli* BW25113 *∆fhuA* was obtained from the Keio collection (strain JW0146) [9]. Liquid growth media used in this study were lysogenic broth (LB), super optimal broth with catabolite repression (SOC), iron-restricted media (IRM; 5.64 g l^−1^ 5x M9 salts, 5 g l^−1^ peptone and 2 g l^−1^ yeast extract were dissolved in 1 l MilliQ water then autoclaved after which 1 M filter-sterile MgSO_4_ was added to a final concentration of 2 mM) or Chelex-100-treated M9 or modified M63 minimal media with reduced phosphate [30.3 mM (NH_4_)_2_SO_4_, 13 mM KH_2_PO_4_, pH 7.0] but no added FeSO_4_. A 1 M MgSO4 solution was added to the defined medium to a final concentration of 1 mM immediately prior to growth experiments. Chelex-100-treated glycerol was added to 0.5% w/v final concentration as a carbon source in minimal medium growth experiments.

For growth curves, an *E. coli* starter culture (1 ml IRM) was grown at 37 °C (14 to 16 h), 150 r.p.m. and used to inoculate 600 µl of growth media in a flat-bottomed 24-well plate (Sarstedt) to a starting OD_600_ of 0.02. Growth curves were performed using a Spectrostar Nano (BMG) at 30 or 37 °C with double orbital shaking (200 r.p.m.) measuring OD_600_ values every 345 s for 24 h. Each strain was grown in triplicate, and the average OD_600_ and standard deviation were reported.

### Whole-genome sequencing of GR536

*E. coli* GR536 was grown in 10 ml LB (37 °C, 150 r.p.m.), pelleted and resuspended in DNA/RNA Shield (Zymo Research), then shipped at room temperature to MicrobesNG (microbesng.com) for whole-genome sequencing. Preparation, sequencing and contig assembly were carried out by MicrobesNG using their standard procedure. Genomic DNA was isolated by lysing cells in TE/lysosome buffer with MetaPolzyme (Sigma-Aldrich, USA) and RNase A (ITW Reagents, Spain), followed by treatment with proteinase K (VWR) and SDS (Sigma-Aldrich). Genomic DNA was purified using SPRI (Solid Phase Reversible Immobilization) beads and resuspended in 10 mM Tris-HCl, pH 8.0. Genomic DNA libraries were prepared using the Nextera XT Library Prep Kit (Illumina) with two modifications: input DNA increased 2-fold and PCR elongation time increased to 45 s. DNA quantification and library preparation were carried out on a Hamilton Microlab STAR automated liquid handling system. Libraries were sequenced on an Illumina sequencer using a 250 bp paired-end protocol. Reads were adapter-trimmed using Trimmomatic version 0.3 (sliding window quality cutoff Q15). *De novo* assembly was performed using SPAdes version 3.7, and contigs were annotated using Prokka 1.11. The assembled genome sequence has been deposited in GenBank (BioProject PRJNA1419173).

### Genome sequence comparison

The GR536 genome sequence was aligned with the *E. coli* W3110 genome sequence [18] using progressiveMauve [19] to identify positions of insertions/deletions and single-nucleotide changes. This information was used to manually identify the nature of the changes in comparison with the W3110 sequence.

### Assays of phage production

Phage production was monitored in growth assays and 50–100 µl aliquots removed from the culture at different OD_600_ values or time points in the early exponential phase, regardless of evidence of lysis. Aliquots were centrifuged for 1 min at 17.7 k × g, and the 40 to 90 µl supernatant was retained. Each aliquot was subject to serial tenfold dilutions, and 4 µl of each dilution was spotted onto 0.6% top agar containing either *E. coli* W3110, BW25113 or BW25113 ∆*fhuA* strains (100 µl of saturated culture per 6 µl top agar) overlaid on LB 1.5% agar. Once spotted, the plates were air-dried (<10 min) and grown at 37 °C for up to 18 h, although plaques became visible after 6 h. Phage titres (p.f.u. ml^−1^) were estimated from plaque counts of the most dilute sample for which clearing was observed.

c.f.u. were determined in parallel using separate aliquots of the same cultures and immediately (without centrifugation) subjected to tenfold dilutions into LB, then plated on LB agar, followed by growth at 37 °C (18–20 h). Colonies (<500 per plate) were counted manually and corrected for dilution to determine c.f.u. of the original culture at the time of harvest.

#### ϕ80 DNA isolation and restriction digestion

Phage DNA was isolated from supernatants or immediately after lysis of cultures growing IRM or from overnight cultures. DNA was purified using a Phage DNA isolation kit (Norgen Biotek Corp.). DNA samples were separately digested with *Bam*HI. Digested DNA fragments were analysed by 1.0% agarose gel electrophoresis.

### Strain manipulations

#### Antibiotic-sensitive GR536

The *kan* and *cat* resistance markers present in GR536 were removed by treatment with plasmid pCP20, as previously described [4, 10]. Antibiotic-sensitive isolates were identified by resuspension of single colonies in 20 µl of LB, followed by spotting 5 µl of the resulting resuspension on LB agar (1.5 %) alone or LB agar (1.5%) plus each antibiotic individually (final concentrations: chloramphenicol, 34 µg ml^−1^, or kanamycin, 50 µg ml^−1^). Antibiotic-sensitive isolates were grown overnight in LB without antibiotics and mixed 1 : 1 with sterile 50% glycerol (v/v). The resulting strain was denoted *E. coli* GR536*.

#### Generation of W3110 lysogen

ϕ80 phage isolated from an overnight culture of *E. coli* GR536 was spotted (5 µl) onto top agar inoculated with *E. coli* W3110 and incubated for 18–20 h at 37 °C. A sterile loop was scraped over the middle of the cleared zone and then used immediately to streak on LB agar for single colonies (18 h, 37 °C). Six single colonies were tested for growth at 37 °C and phage production using a top agar assay. Positive isolates were grown overnight in LB without antibiotics and mixed 1 : 1 with sterile 50% glycerol (v/v) for long-term storage.

### Construction of pBAD30 ∆MCS

Deletion of the multiple cloning site of pBAD30 was carried out in three steps. The purified plasmid was first digested with *Eco*RI and *Hin*dIII. The digestion mixture was separated on a 1% agarose gel, and the resulting linear fragment (4.9 kb) was excised from the gel and purified using a Monarch^®^ DNA Gel Extraction Kit (New England Biolabs, Inc). The purified fragment was then incubated with T4 DNA polymerase (New England Biolabs, Inc) and dNTPs for 15 min at 12 °C. The reaction mixture was cleaned up using Monarch^®^ PCR and DNA Cleanup Kit (New England Biolabs, Inc), and the purified blunt-ended fragment was incubated with T4 DNA ligase (20 °C, 16 h), and the reaction mixture was transformed into chemically competent *E. coli* XL-1 Blue. Plasmid DNA was purified from three colonies (grown overnight in 1.5 ml LB) and digested with *Eco*RV and *Eco*RI or *Sal*I or with pBAD30 as a positive control (for intact *Eco*RI and *Sal*I restriction sites).

## Results

### GR536 growth

**Fig. 1.**
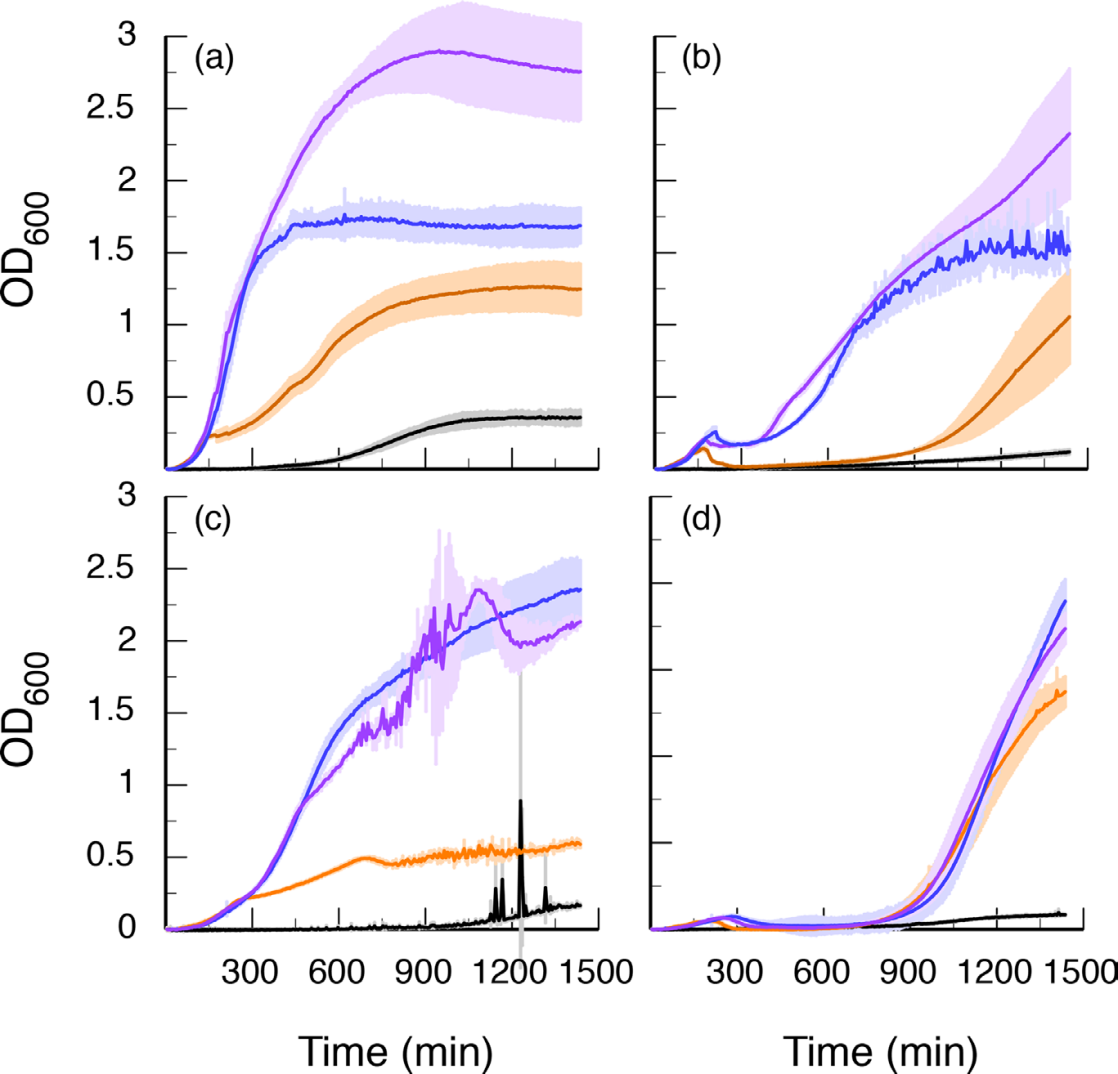
Apparent lysis of GR536 in different growth media. Cells (GR536 or GR536+pBAD30) were grown in modified M63 (black line), IRM (orange line), SOC (blue line) and LB (purple) with no added antibiotic. (a, c) GR536 at 37 °C and 30 °C. (b, d) GR536+pBAD30. Triangles (▲) indicate time points (180 and 210 min) at which 50 µl aliquots were removed from 37 °C cultures to determine phage titre. All curves are averages of three cultures.

Prior to its use to test the activity of a heterologously expressed metal transporter system, we tested GR536 growth in an iron-deficient medium (IRM; Fig. 1). We noticed unstable growth in this medium with an empty-vector control (pBAD30) [14], indicated by a decrease in growth rate at OD_600_~0.15 (~180 min) consistent with cell lysis rather than cessation of growth. This effect was less obvious in the GR536 strain without plasmid, but with growth still slowing in the early exponential phase (Fig. 1). In iron-replete media (LB and SOC), GR536 grew without obvious perturbation; however, lysis was still apparent when pBAD30 was present (Fig. 1). Neither strain grew well in the first 10 h in Chelex-treated defined minimal media without supplemental iron (modified M63), although GR536 did show growth after this point. In strains with limited growth, or growth post-lysis, growth eventually resumed, raising the possibility of a suppressor mutation or some other genetic change restoring viability. Similar behaviour for this strain has not been previously reported, although differences in liquid media choice, endpoint-only growth measurements or use of log-scale to plot data may have precluded detection of the observed atypical growth behaviour in early exponential phase [4–6].

### GR536 genome sequence features

To determine if the growth behaviours of *E. coli* GR536 were solely due to the deletion of key metal-uptake genes or a result of genetic change(s) during the creation and passaging of the strain prior to transfer to our labs, we performed whole-genome sequencing to verify the strain genotype. The assembled genome (4,669,703 bp; 50.81% GC; Bioproject PRJNA1419173) was aligned with *Escherichia coli* W3110 (BioProject PRJNA16351). The whole-genome sequence revealed the expected deletions to the metal-uptake genes (Table 1) and the presence of the *kan* and *cat* antibiotic resistance markers in the *fecAE* operon and *zupT* gene, respectively, compared to the parent strain [18]. The locations of the FLP-recombinase sites that enabled successive rounds of excision of the *cat* antibiotic resistance marker from within the disrupted *entC*, *mntH* and *zupT* genes were all confirmed. A combined additional 23 insertions or deletions (Table 1), including 9 IS element deletions and 3 IS element insertions and 7 other insertion or deletion events, as well as 74 individual nucleotide changes (Table S2, available in the online Supplementary Material), were also present.

**Table 1.** Nucleotide insertions and deletions observed in *E. coli* GR536 compared to *E. coli* W3110

GR536 location*	Change in GR536	W3110 location*	Notes
146605–147833	*IS2* insertion	3903280	*bax disruption*
202776	34 bp deletion in *dctA*	3958123–3958156	Truncated DctA (390 aa vs 428 aa)
342070–342153	***∆feoABC deletion***	4097445–4100172	Scar from *cat* removal†
552344	*IS5* deletion	4310363–4311451	*yjcS alsK* intergenic region
571542	72 bp deletion	4330755–4330826	*phnA phnB* intergenic region
756063–757538	**∆*fecABCDE disruption***	4515470–4521238	*kan* cassette inserted†
1430473	1 nt insertion (agg**g**gga)	547835	Restores *ylbE* reading frame
1506696–1506899	***∆entC* deletion**	624171–625204	Scar from *cat* removal†
1535916	*IS5* deletion	654214–655407	Restores *dcuC*
1898129	26 bp deletion in *ycbZ*	1017631–1017656	tcggtcattgaatttccgggtcatcc
1978457–1978637	2×181 bp insertion	1097805	*serX ycdU* intergenic region‡
2078784	Excised e14 prophage	1197952–1212989	Restores *icd*
2166668	*IS5* deletion	1301041–1302370	*ychE oppA* intergenic region
2176594–2222714	**ϕ80 phage insertion**	1312283–1312309	*attB* site within 3′ end of *yciI*§
2561189–2561353	Orf insertion (6.3 kDa)		Between *ydcF* and *dicF*
3088751	*IS5* deletion	2176984–2178176	Restores *gatA*
3231306	*IS2* deletion	2320739–2322124	Restores *rcsC*
3426216–3426269	**∆*mntH* deletion**	2516948–2518121	Scar from *cat* removal†
3472298–3479079	CPZ-55 prophage	3472298	Between *eutB* and *eutA*
3515392–3156729	*IS186* insertion	2600452	Disrupts *hyf*
4097541–4098563	***∆zupT* disruption**	3181244–3181948	*cat* cassette inserted†
4177745	*IS5* deletion	3261134–3262328	Restores *tdcD*
4428177–4429507	*IS3* insertion	3512759	Disrupts *cytR*
4514042–4514048	*rrlA* 2 bp deletion	3597289–3597294	*gc**c**gcaa**g**g to gcgcaag*
4659688	*IS2* deletion	3742396–3744263	*yidZ-yieE* intergenic region
4662321	*IS5* deletion	3746911–3748105	Restores *tnaB*
4664802	*IS5* deletion	3750591–3751785	*tnaA tnaC* intergenic region

*Number range indicates deletion of this region in the observed other strain (single number indicates nucleotide at which deletion begins).

†Scar nucleotide sequence can be found in Fig. 3, [10].

‡tacgaaaaaaaagcccgtactttcgtacgagctcttctttaaatatggcggtgagggggggattgactcgcttcgctcgccctgcgggcagcccgctcactgcgttcacggtctgtccaactggctgtcgccagttgtcgaaccccggtcggggcttctcatccccccggtgtgtgcaata.

§ɸ80 *attB* sequence: gtgaaaccatttaagaaagtgttctga.

Notably, we identified a 46.15 kbp insertion (location) that corresponded to the ϕ80 prophage [13, 20] at its canonical integration site. The prophage was identified as ϕ80 by a blastn search, using the full 46.15 kbp insertion copied from the GR536 sequence, and showed 100% identity to the published ϕ80 sequence [12]. A second cryptic phage, CPZ-55 [21], was also present in the GR536 genome, when compared to W3110. CPZ-55 is present in *E. coli* BW25113 [22, 23], although this strain was not used in the construction of GR536. Another cryptic prophage, e14 [21, 24], was absent from GR536 when compared to W3110. Its presence or absence in *E. coli* BW25113 may affect strain fitness under some growth conditions [22]. Of the remaining insertions, deletions or nucleotide changes, none seemed likely to be responsible for the observed growth behaviour, making ϕ80-dependent lysis the most likely cause of the unexpected growth behaviour.

### Evidence for ϕ80 production in early exponential phase growth

ϕ80 is a lambdoid phage that switches to the lytic cycle in an SOS-dependent manner and may be widely present in laboratory strain collections due to its interference with P1 phage life cycle and its reduced lytic capability at 37 °C [11]. To test whether ϕ80 was responsible for the apparent lysis of GR536, both with and without pBAD30, we collected aliquots of the growth media at 180 and 210 min after the start of growth (Fig. 1) and determined phage titre using a plaque assay with W3110 as the recipient strain (Fig. S1). Nearly all strain and growth media combinations showed evidence of phage production, with the highest titres observed for GR536+pBAD30 in IRM and LB (~4×10^9^ p.f.u. ml^−1^) compared to 10^3^–10^4^-fold lower values for GR536 alone. Little phage production (titre) was observed in minimal reduced phosphate medium, which likely reflected the slower growth and low cell density at the collection times (Fig. 1).

**Fig. 2.**
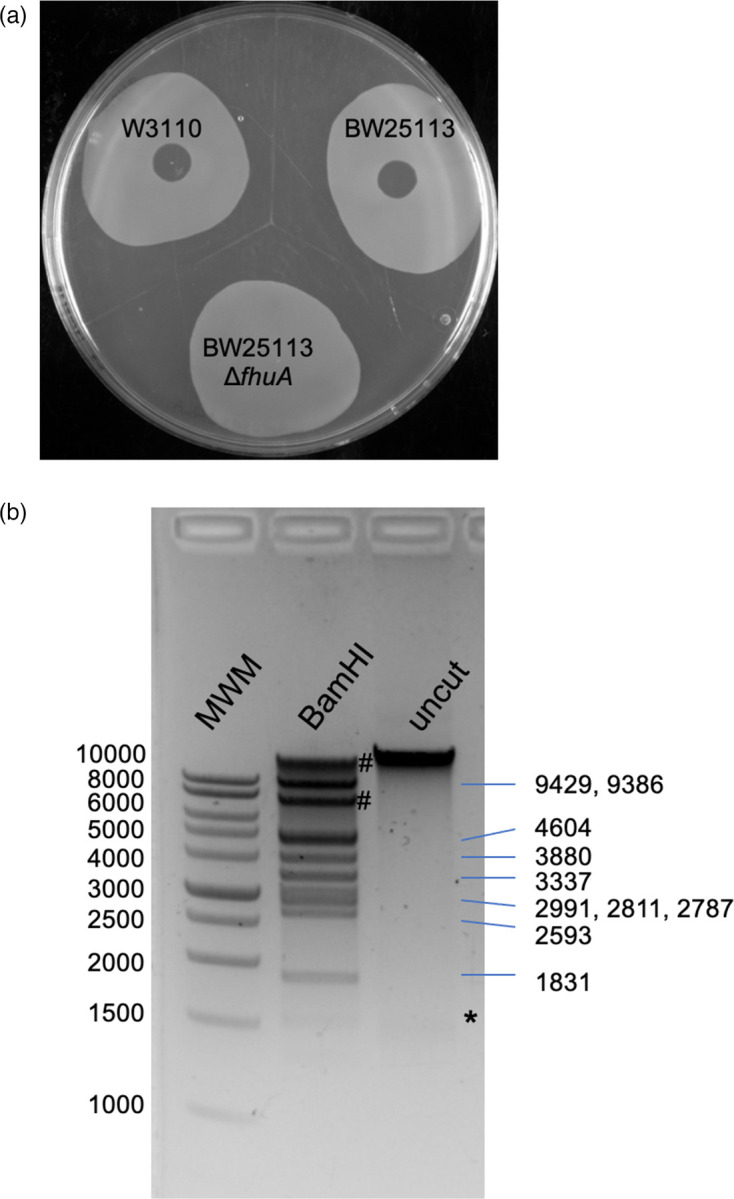
Identification of ϕ80 as the phage produced by *E. coli* GR536. (a) Top agar assay of GR536 media supernatants spotted on different *E. coli* strains, as indicated in the image. (b) *Bam*HI restriction digestion of purified phage DNA. Phage genomic DNA was digested with BamHI as described in the text. * indicates a band present in both uncut and cut samples, and # indicates bands of >10,000 and ~7,500 bp that do not correspond to any fully digested *Bam*HI fragment.

The identification of ϕ80 as the phage produced was tested in two ways. First, the phage samples were plated on top agar containing *E. coli* BW25113 *∆fhuA*, a strain deleted for the ϕ80 receptor (FhuA) [25, 26]. The phage produced by GR536 did not produce any plaques when spotted on top agar containing the ∆*fhuA* strain but did clear the top agar containing W3110 and BW25113 strains (Fig. 2). To further support the identity of ϕ80 as the isolated phage, we purified phage DNA from a culture after overnight growth, then performed a *Bam*HI restriction digest, as used previously for analysis of ϕ80 [12]. The observed digest pattern is consistent with the presence of ϕ80 DNA based on observed band sizes (Figs 2 and S2). However, some additional bands were observed (>10,000 bp, <8,000 bp and a more intense band >5,000 bp, suggesting more than one band of size ~4,600 bp). These unexplained bands may correspond to partially digested 80 DNA, as they are not consistent with the sizes of *Bam*HI fragments of the other cryptic prophages present in GR536. Similar to this work, not all expected *Bam*HI bands were observed in the previous study [12] (~10 of 14 expected bands) and deletion of a predicted ϕ80-encoded DNA methylase (*damL*) in that work resulted in an altered *Bam*HI digest pattern, suggesting that *Bam*HI site cleavage (GGATCC) may be affected by the phage-encoded methylase. Nonetheless, the presence of expected band sizes and the inability to infect the ∆*fhuA* strain are consistent with ϕ80 production by GR536.

### Factors affecting GR536 lysis by ϕ80

**Fig. 3.**
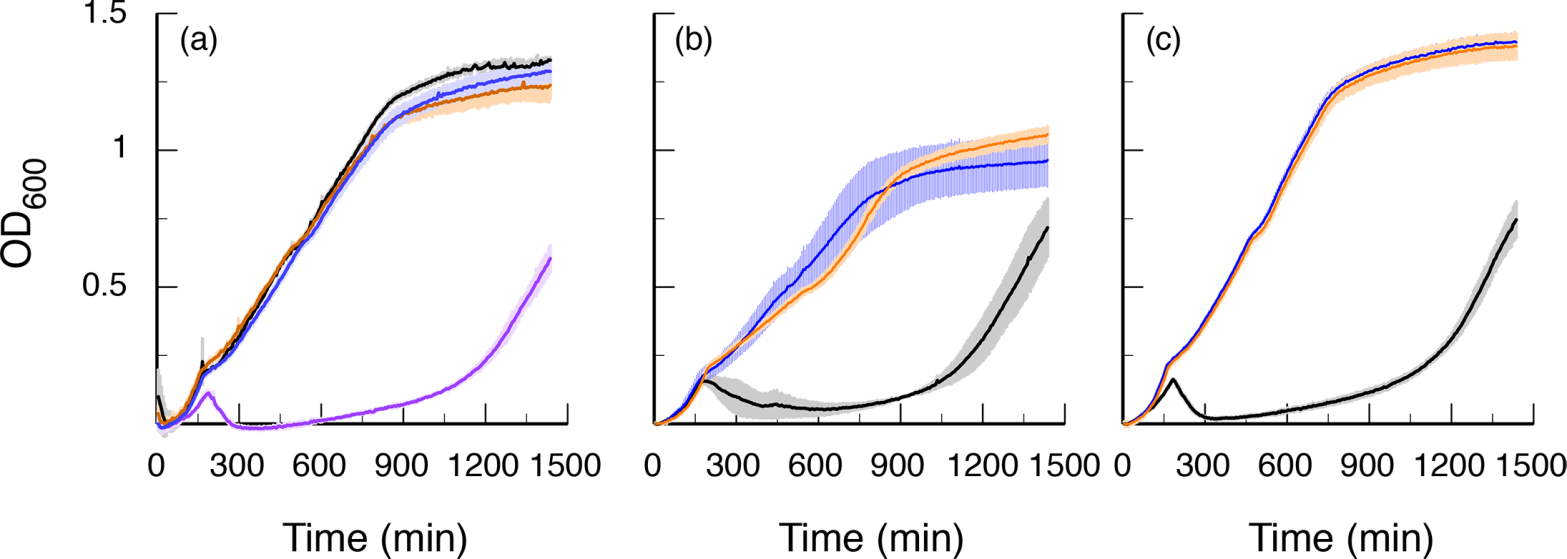
pBAD30 interacts negatively with *E. coli* GR536. *E. coli* strains GR536 and GR536* without and with different plasmids were grown in IRM medium (37 °C, 200 r.p.m., initial OD_600_ 0.02). (a) GR536 (black), GR536* (orange), GR536+pBAD30 (purple) and GR536*+pBAD30 (blue). (b) GR536+pBAD30 (black), GR536+pUC18 (blue) and GR536+pMPM-A5 (orange). (c) GR536+pBAD30 (black), GR536+pBAD30 ∆MCS (blue) and GR536+pMncA (orange). Growth medium for experiments in which all strains contained a plasmid (panels b and c) contained ampicillin (100 µg ml^−1^ final concentration). All curves are averages of three cultures.

To better understand the ϕ80-dependent lysis of GR536 observed here in comparison to previous studies, we tested different potential contributing factors. A previous study that used GR536 to test candidate Fe-importer genes (*efeUOX*) using plasmid-based complementation had two key differences from our experiments, namely the removal of the *cat* and *kan* resistance markers (to generate strain JC28) and the identity of the transformed plasmid [4]. We first generated the antibiotic-sensitive strain, here named *E. coli* GR536*, in the same way as that study, and observed no obvious difference in growth between the two strains (Fig. 3a) without pBAD30. However, a significant reduction in lysis was observed for *E. coli* GR536* containing pBAD30, compared to *E. coli* GR536 with pBAD. This difference suggested a negative interaction between one or both of the resistance markers and some component of pBAD30.

We then tested whether other plasmids, pUC18 [15] and pMPM-A5 [16], would affect *E. coli* GR536 growth in the same way as pBAD30 and observed that only pBAD30 caused the marked increase in lysis and phage production (Fig. 3b). Notably, pMPM-A5 is a pBAD-based expression system, suggesting that the promoter itself is not a cause of the negative interaction. Surprisingly, a pBAD30 construct with a gene inserted into the multiple cloning region (pBAD30-*mncA*) [17] also showed a reduction in apparent lysis compared to pBAD30 (Fig. 3c). This construct encodes a cytosolic metal-binding protein (MncA) that, when expressed in *E. coli*, irreversibly binds Fe(II) [17] and thus would not be expected to support improved growth in the metal-uptake-deficient *E. coli* GR536 compared to pBAD30 without any insert.

The combination of the removal of the resistance markers (GR536*) and the apparent sensitivity of *E. coli* GR536 to pBAD30 but not other plasmids, or a version of pBAD30 with a gene inserted in the MCS, suggested that our original observation of lysis was in fact due to a negative interaction between one or both of the antibiotic resistance markers (or nearby gene) and the multiple cloning site of pBAD30 by some unknown mechanism. We tested this latter possibility by deleting the multiple cloning site of pBAD30 (pBAD-∆MCS; Figs S3 and S4) and observed substantially reduced lysis and phage production compared to the parent plasmid (Fig. 3c). The specific molecular basis for the negative interaction was not investigated further, although possibilities are discussed later. Critically, these observations indicate that use of pBAD30 as a control vector in GR536 that retains the *cat* and *kan* resistance markers will yield a false-positive result compared with pBAD30 containing any inserted gene because the insertion will apparently relieve the mechanism that leads to lysis by ϕ80.

### Loss of metal-uptake genes does not increase ϕ80 production upon switch to lytic growth

**Fig. 4.**
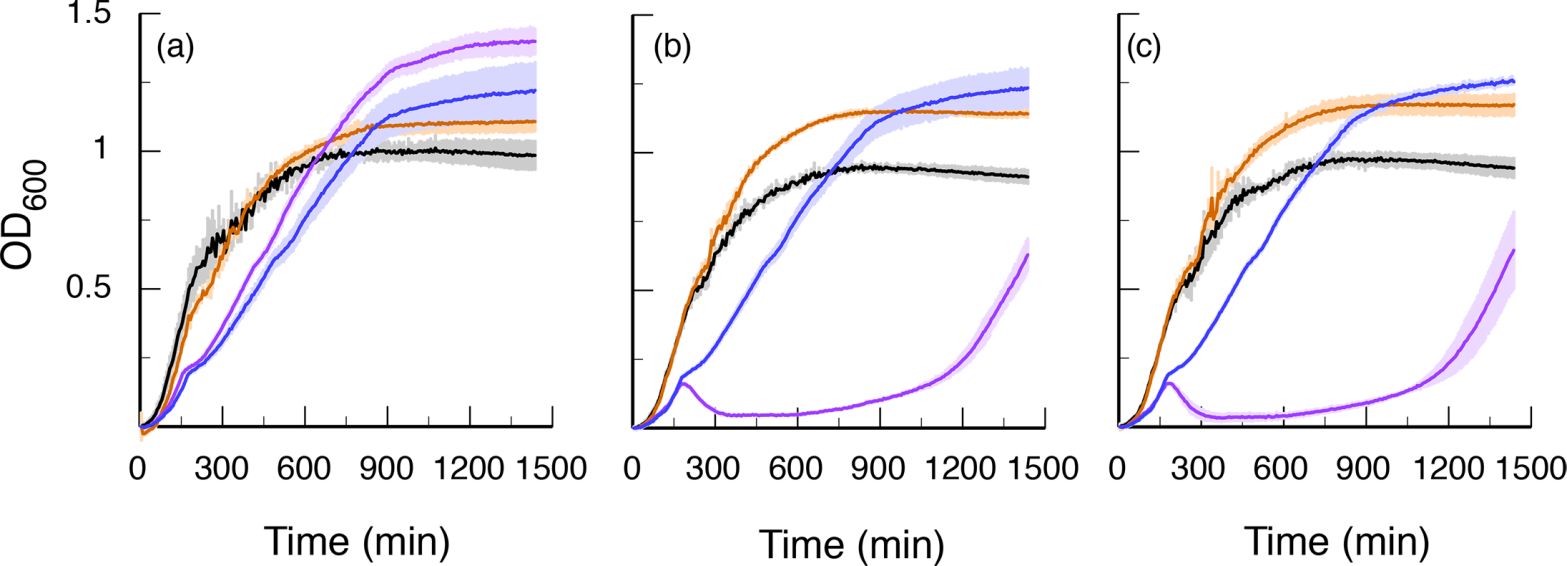
Comparison of growth between *E. coli* lysogens containing ϕ80 prophage. Cells were grown in IRM medium (37 °C, 200 r.p.m., OD_600_ initial 0.02). (a) W3110 (black), W3110 lysogen (orange), GR536* lysogen (blue) and GR536 lysogen (purple). (b) Each strain transformed with pBAD30 (no antibiotic added during growth). (c) Each strain transformed with pBAD30 with 100 µg ml^−1^ ampicillin added to growth medium. All curves are averages of three cultures.

To assess the effects of the multiple metal-uptake gene deletions in GR536 on ϕ80-dependent lysis and phage particle production, we generated a W3110 lysogen containing the ϕ80 prophage (Fig. S5). The W3110 lysogen showed very subtle differences in growth compared to the W3110 parent (Fig. 4) but still produced a large titre of phage (Fig. S6a; 7.0×10^9^ p.f.u. ml^−1^). Phage production by GR536 was comparable (1.6×10^10^ p.f.u. ml^−1^) to the W3110 lysogen, while, in contrast, GR536* showed up to 10^4^-fold less phage production (2.0×10^6^ p.f.u. ml^−1^). ϕ80 production had a negligible effect on cell viability (Fig. S6b) in the W3110 lysogen compared to the parent strain (2.1×10^8^ c.f.u. ml^−1^ versus 1.9×10^8^ c.f.u. ml^−1^). In contrast, ϕ80 production reduced viability in both GR536 and GR536* (<10^5^ c.f.u. ml^−1^ and 1.9×10^7^ c.f.u. ml^−1^), with lysis likely occurring at the time of sampling and during serial dilution for both strains, as sampling occurred before any obvious change in OD_600_. Thus, while the effect of ϕ80 production on GR536 growth may be more obvious than for the W3110 lysogen based on OD_600_, the phage titre is comparable. Comparison of the W3110 and GR536* lysogens indicates that the metal-uptake deficiency does not contribute to increased phage production (10^4^-fold lower in GR536*). Instead, it is the metal-independent negative interaction between pBAD30 and one or both of the antibiotic resistance markers in the GR536 background.

## Discussion

We have shown that *E. coli* GR536 contains the ϕ80 prophage and have identified conditions where lysis is particularly likely to be observed. Based on the absence of any genetic manipulation since receiving the strain, we assume that the prophage was either introduced during strain construction or was present in the parent strain given its reported stealthy nature [11, 12]. The process of creating GR536 involved stepwise generation of individual knockouts in the original W3110 background (GR417, ∆*entC::cat*; GR460, ∆*feoABC::cat* ∆*entC*; GR489, ∆*mntH::cat* ∆*feoABC* ∆*entC*; GR499, ∆*zupT::cat* ∆*mntHt* ∆*feoABC* ∆*entC*; and GR536, ∆*fecABCDE::kan* ∆zupT::*cat* ∆*mntH* ∆*feoABC* ∆*entC*) [3]. The *cat* resistance marker was removed after the creation of each of the first three strains in the series. The presence of ϕ80 has been considered hard to detect because of its high frequency of lysogeny (up to 100-fold higher than λ phage) when the phage is present in an *E. coli* culture or colony, meaning a substantial fraction of cells continue growing, making lysis harder to detect [11]. Additionally, at typical *E. coli* growth temperatures (37 °C), ϕ80 is much less likely to convert to lytic growth than λ, further limiting its observation [11]. Previous analysis of ϕ80 growth behaviour suggests that this phage is particularly adept at hijacking P1 phage particle assembly [11]; however, P1 transduction as a source of the prophage in this case is ambiguous, as the description of the construction of GR536 is not sufficiently clear to confirm its use [3]. More likely, the prophage was present in the parent strain and, for the reasons mentioned above, hard to detect. Thus, the point of the appearance of the ϕ80 prophage in the strain lineage culminating in GR536 cannot currently be identified but could be determined by analysis of those preceding strains for ϕ80 production.

The timings of the numerous changes in other mobile genetic elements – the appearance of the CPZ-55 prophage, loss of the e14 prophage and the net loss of 6 IS elements – are also unclear. In principle, the CPZ-55 prophage could potentially have been transferred via P1 transduction from a strain in which it was already present, as it is within ~46 kb of the *mntH::cat*, but as P1 transduction was not obviously used otherwise, it was likely present, but this prophage may also have been present in the lab stock of W3110 used for the construction of GR536 and its precursors. Likely causes that explain the changes in IS elements are also speculative, ranging from the repeated use of the λ *red* (on plasmid pKD46) and FLP recombinases (on plasmid pCP20) to insert and excise the antibiotic resistance cassettes necessary for strain construction to the reduced fitness of the strains upon loss of metal-uptake systems. Genome sequences of the strains listed earlier would clarify when these events occurred.

Our choice of pBAD30 as an expression vector enabled identification of the presence of the ϕ80 prophage in *E. coli* GR536 because of its adverse interaction with the strain (10^4^-fold increase in pfu/ml compared to *E. coli* GR536*). The specific molecular basis for the negative interaction between GR536 and pBAD30 that leads to elevated ϕ80 phage production was not determined in this study but may be an idiosyncratic consequence of this strain and plasmid combination. The correlation between the presence of the intact pBAD30 MCS and elevated phage titres suggests that a specific aspect of the DNA sequence contributes to increased phage production. There are likely three formal possibilities for how this sequence might generate a negative interaction. Prediction of open reading frames encoded by intact pBAD30 identified a hypothetical ORF (92 amino acids) expressed in the opposite (complementary) direction to any gene expressed under the control of P*_BAD_* (Fig. S4). A tblastn search using this sequence showed that the predicted ORF is encoded by numerous pBAD-derived plasmids, all of which would have been derived from pBAD18, in which the MCS and terminator sequence combination was first introduced [14], and not homologous to any widely abundant genome-encoded proteins. Similarly, AlphaFold does not predict any structure for this ORF. If a polypeptide was expressed, it would need to interact with some feature of GR536, but not GR536*. The other two formal possibilities include the MCS DNA sequence of the MCS or a transcript spanning this region interacting with a molecule present in GR536 but not GR536*. In all three cases, the interaction would likely have to increase endogenous DNA damage to result in increased phage titres [12]. We note for completeness that pKD46 [10], used in strain construction, is based on pBAD18 but would lack an intact MCS.

There appear to be two possibilities for the genetic contribution from GR536 that contributes to increased phage production when pBAD30 is present in the strain. As removal of the antibiotic resistance genes relieved the negative interaction with the plasmid, the causes must be linked to the consequences of the insertion of these genes in the *E. coli* chromosome. The *zupT* gene is found in the opposite orientation between *ygiD* (116 bp between the start of each gene) and *yqiD* (58 bp between the end of each gene). The *cat* resistance marker that disrupts *zupT* is in the same orientation as both *ygiD* and *yqiD*, so it could potentially have a polar effect on the expression of *ygiD*, in particular. YgiD is annotated as a 4,5-DOPA dioxygenase [27], which seems unlikely to be the cause of the negative interaction. In contrast, the *kan* cassette fragment, amplified using the pKD4 template plasmid [10], appears capable of expressing an additional 81 amino acid ORF that is predicted to encode a stably folded domain (Fig. S7), which is further fused to the complementary sequence of the remaining portion of the 5′ region of the *fecA* gene, resulting in a hypothetical 159 amino acid ORF, although AlphaFold did not predict a structure for this sequence, which had no homologues in a BlastP search. We note that the cassette-encoded ORF in pKD4 is not present in the pKD13 template, which was used to generate the Keio collection of *E. coli* single-knockout mutants via *kan* cassette gene disruption [9]. Owing to the multiple steps required to test the specific hypothesis that a *kan* resistance cassette-encoded ORF interacts negatively with pBAD30, it would require multiple steps – generation of an *E. coli* lysogen and then insertion of a pKD4-derived *kan* cassette into at least two loci or vice versa – and then testing phage production in the presence and absence of pBAD30. This approach would discriminate between a general feature of the *kan* resistance cassette (the X-aa ORF) and a very specific feature of the fusion protein created by the insertion of the resistance cassette at a precise location within the *fecA* gene (encoding the Xx-aa ORF).

Finally, the presence of the ϕ80 prophage in the *E. coli* GR536 lysogen is not an impediment for using it to study metal transport, particularly as pBAD30 was not used as a control plasmid [3, 4, 7]. Removal of the antibiotic resistance markers to create GR536*, or the (assumed) otherwise genetically identical JC28 [4], reduces phage production relative to both GR536 and the W3110 lysogen, the latter of which contains a full set of metal-uptake genes, thus indicating that phage production is not increased as a consequence of the impaired metal homeostasis in GR536*. The observation of ϕ80-dependent lysis under specific growth conditions may require continuous monitoring of optical density (every 6 min in this work), particularly in early exponential growth (OD_600_ ≤0.25). Previous studies of growth have used less frequent timepoints (≥60 min) [4, 5] or end-point growth (12 to 16 h) [3, 6], in which case effects of lysis on growth would be much harder to detect. Further studies will be needed to determine whether complementation of *E. coli* GR536* (or JC28) with a functional Fe-uptake gene cluster alters phage production, perhaps increasing ϕ80 production as suggested by the high phage titres of the W3110 lysogen.

## Supplementary material

10.1099/acmi.0.001204.v3 Supplementary Material 1.

## Abbreviations

IRM: iron-restricted media
LB: lysogenic broth
SOC: super optimal broth with catabolite repression
