# Supplementary material for: The metal-uptake-deficient Escherichia coli strain GR536 contains the ϕ80 prophage
Source: Access Microbiol. 2026 Aug 7;8(8):001204.v3. doi: 10.1099/acmi.0.001204.v3 (PMC13449921; doi:10.1099/acmi.0.001204.v3)
Supplement: Supplementary file 1 [file acmi-8-01204-s001.pdf]

Supplementary Information

The metal-uptake-deficient *Escherichia coli* strain GR536 contains the ϕ80 prophage

Ayuki Shimpo[1], Jerry Augustine[1], Luke J. Acton[3], Lindsay J. Hall[2,3], Peter T. Chivers[1,*]

[1]Department of Biosciences, Durham University, Durham, UK DH1 3LE

[2]Department of Microbes, Infection and Microbiomes, School of Infection, Inflammation and Immunology, College of Medicine and Health, University of Birmingham, Birmingham, UK, B15 2TT

[3]Institute of Microbiology and Infection, University of Birmingham, Birmingham, UK B15 2TT

*Corresponding author: peter.chivers@durham.ac.uk

List of Supplementary Figures and Tables

Table S1. SNPs present in *E. coli* GR536 compared to *E. coli* W3110.

Figure S1. GR536 releases high titres of ϕ80 in early log phase.

Figure S2. Map of ϕ80 genome showing *Bam*HI restriction sites.

Figure S3. Restriction digestion screen of candidate pBAD30 ΔMCS constructs.

Figure S4. Plasmid maps of pBAD30 and pBAD30 ΔMCS.

Figure S5. Screening for W3110 lysogens.

Figure S6. Phage production by different strains containing pBAD30 results in different amounts of cell lysis.

Figure S7. Identification of a hypothetical 159 amino acid ORF fusion resulting from the *kan* cassette disruption of *fecABCDE*.

Table S1 SNPs present in *E. coli* GR536 compared to *E. coli* W3110

SNP	W3110 Position	GR536 position	Notes
AG	224772	1107410	*rrsH*
CG	224776	1107414	*rrsH*
CT	224780	1107418	*rrsH*
GA	224789	1107427	*rrsH*
AG	224790	1107428	*rrsH*
TA	224791	1107429	*rrsH*
TA	224792	1107430	*rrsH*
GT	224793	1107431	*rrsH*
TC	224808	1107446	*rrsH*
TC	224890	1107528	*rrsH*
AG	547694	1430332	*ylbE*
AT	556858	1439497	*folD*
GT	987574	1868078	*ompF-asnS intergenic*
TC	1093686	1974156	*ycdT*
CT	1097580	1978050	*ycdU-serX intergenic*
CT	1197797	2078629	*icd prophage*
CT	1197809	2078641	*icd prophage*
GT	1519222	2429652	*ydcX*
GT	1979957	2891722	*flhD-ins intergenic*
CT	2005401	2917166	*RS10120*
AG	2245455	3156021	*yeiE*
TC	2726106	3642398	*rrlG rRNA*
CG	2726107	3642399	*rrlG rRNA*
CA	2726108	3642400	*rrlG rRNA*
GA	2726111	3642403	*rrlG rRNA*
GT	2726114	3642406	*rrlG rRNA*
GC	2726115	3642407	*rrlG rRNA*
AG	2726116	3642408	*rrlG rRNA*
CT	2726118	3642410	*rrlG rRNA*
AG	2866111	3782403	*ftsB*
CG	3181243	4097535	*ygiE*
AC	3181244	4097536	*ygiE*
CT	3181246	4097539	*ygiE*
CT	3181247	4097540	*ygiE*
AG	3181248	4097541	*ygiE*
CT	3181249	4097542	*ygiE*
TA	3181252	4097545	*ygiE*
TG	3181253	4097546	*ygiE*
AG	3181254	4097547	*ygiE*
TC	3181255	4097548	*ygiE*
AG	3268825	4184242	*yhaC*
TA	3424507	4339924	*rrfD*
AT	3426661	4342078	*intergenic*
TC	3426946	4342363	*intergenic*
TC	3466306	4381723	*sugE*
CG	3466307	4381724	*sugE*
CA	3466308	4381725	*sugE*
GA	3466311	4381728	*sugE*
GT	3466314	4381731	*sugE*
GC	3466315	4381732	*sugE*
AG	3466316	4381733	*sugE*
CT	3466318	4381735	*sugE*
AG	3597953	4514704	*rsmD*
AG	3597986	4514737	*rsmD*
TC	3597992	4514744	*rsmD*
AC	3600947	4517699	*zntA*
CT	3690767	4607519	*rrlC rRNA*
CA	3690775	4607527	*rrlC rRNA*

GA	3691113	4607865	*rrlC rRNA*
AG	3692621	4609373	*rrlC rRNA*
TC	3692622	4609374	*rrlC rRNA*
TC	3692693	4609445	*rrlC rRNA*
TC	4005879	250496	*yhiM-yhiL intergenic*
CT	4019331	263948	*rhsB*
GA	4215355	457342	*rrl rRNA*
TC	4215357	457344	*rrl rRNA*
CG	4215358	457345	*rrl rRNA*
CA	4215359	457346	*rrl rRNA*
CT	4215362	457349	*rrl rRNA*
GT	4215365	457352	*rrl rRNA*
GC	4215366	457353	*rrl rRNA*
AG	4215367	457354	*rrl rRNA*
AG	4215888	457875	*rrl rRNA*
GA	4305725	547712	*yicO*

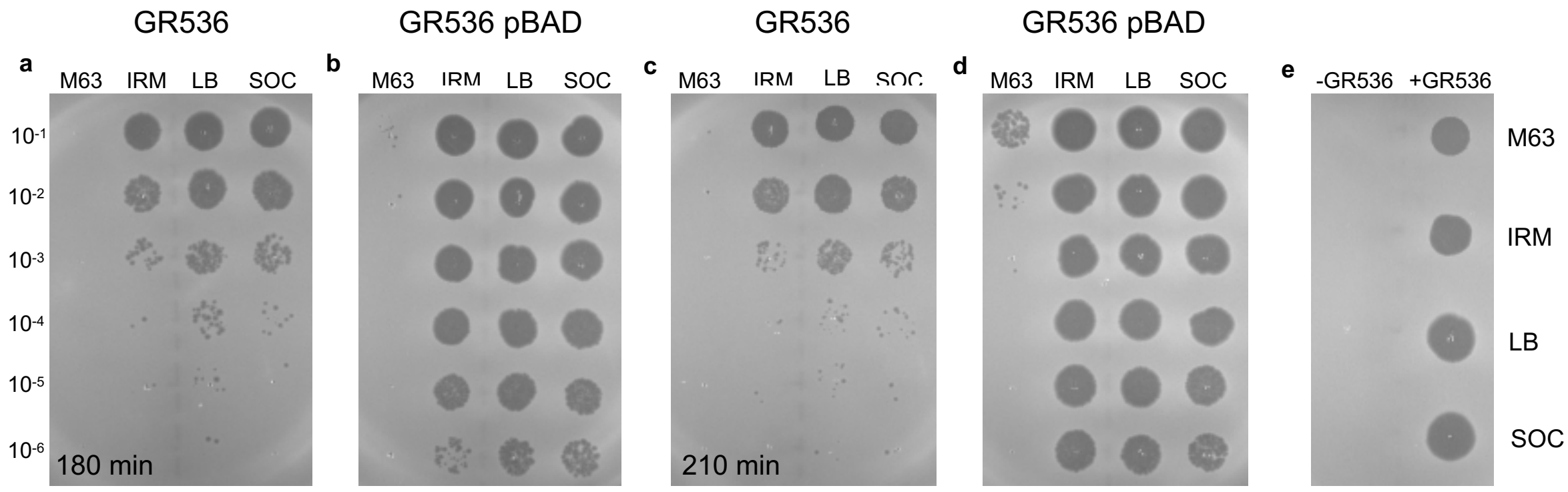

Figure S1. **GR536 releases high titres of f80 in early log phase**. Culture supernatants were applied to top agar containing *E. coli* W3110 as described. a and c, phage titres of GR536 at 180 and 210 min. Panels b and d, phage titres of GR536 + pBAD at 180 and 210 min. e, different undiluted media versus undiluted GR536 supernatant after 16 h growth.

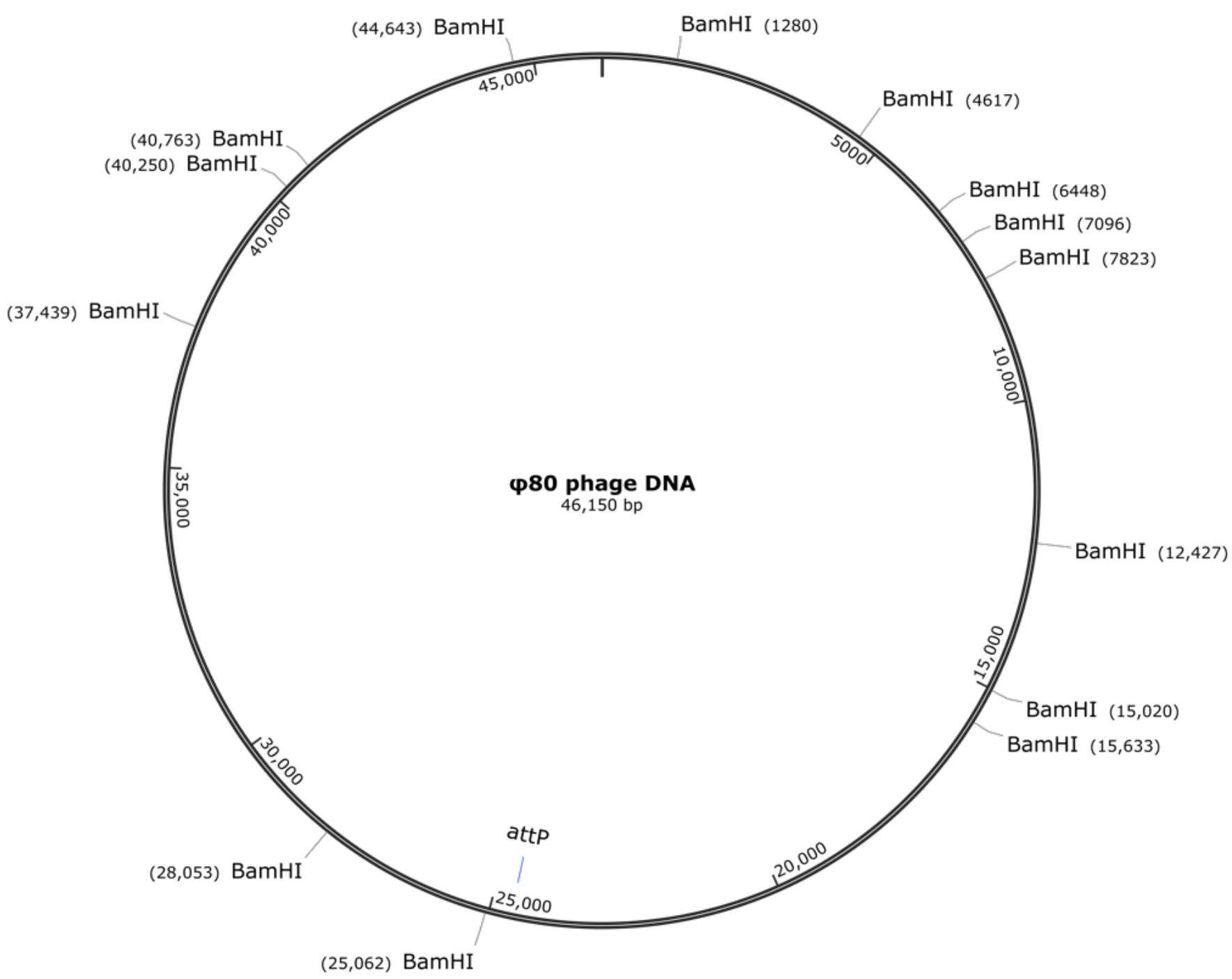

9429
9386
4604
3880
3337
2991
2811
2787
2593
1831
727
648
613
517

Figure S2. Map of ϕ80 genome showing *Bam*HI restriction sites. The linear ϕ80 nucleotide sequence was copied from the full *E. coli* GR536 sequence and circularised using SnapGene. A total of *Bam*HI 14 fragments are predicted from a *Bam*HI restriction digest of circular phage DNA. The position of the ϕ80 *attP* sequence is shown on the map (24623 - 24639 bp).

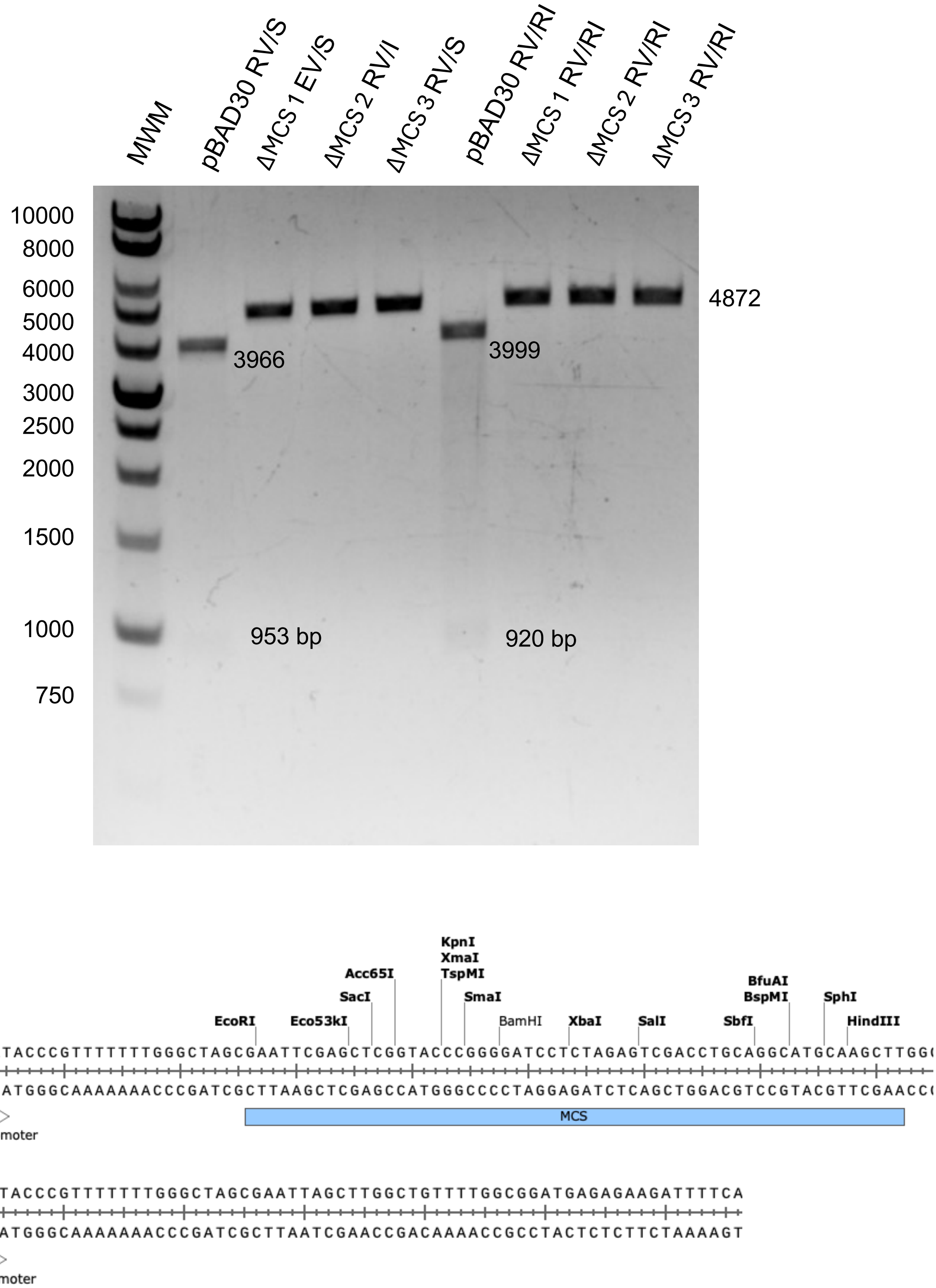

Figure S3. Restriction digestion screen of candidate pBAD30 ∆MCS constructs. Candidate plasmids were digested with *Eco*RV (RV) and *Sal*I (S) or *Eco*RV and *Eco*RI (RI). The *Sal*I and *Eco*RI sites should not be present in the ∆MCS construct and the plasmid will show only a single cut by *Eco*RV, compared to the parent plasmid (pBAD30), which will have two bands. Molecular weights of the expected bands are shown on the image. Nucleotide sequences of the pBAD30 MCS and ∆MCS regions are shown below the gel image. Plasmid maps of both constructs are shown in Figure S4.

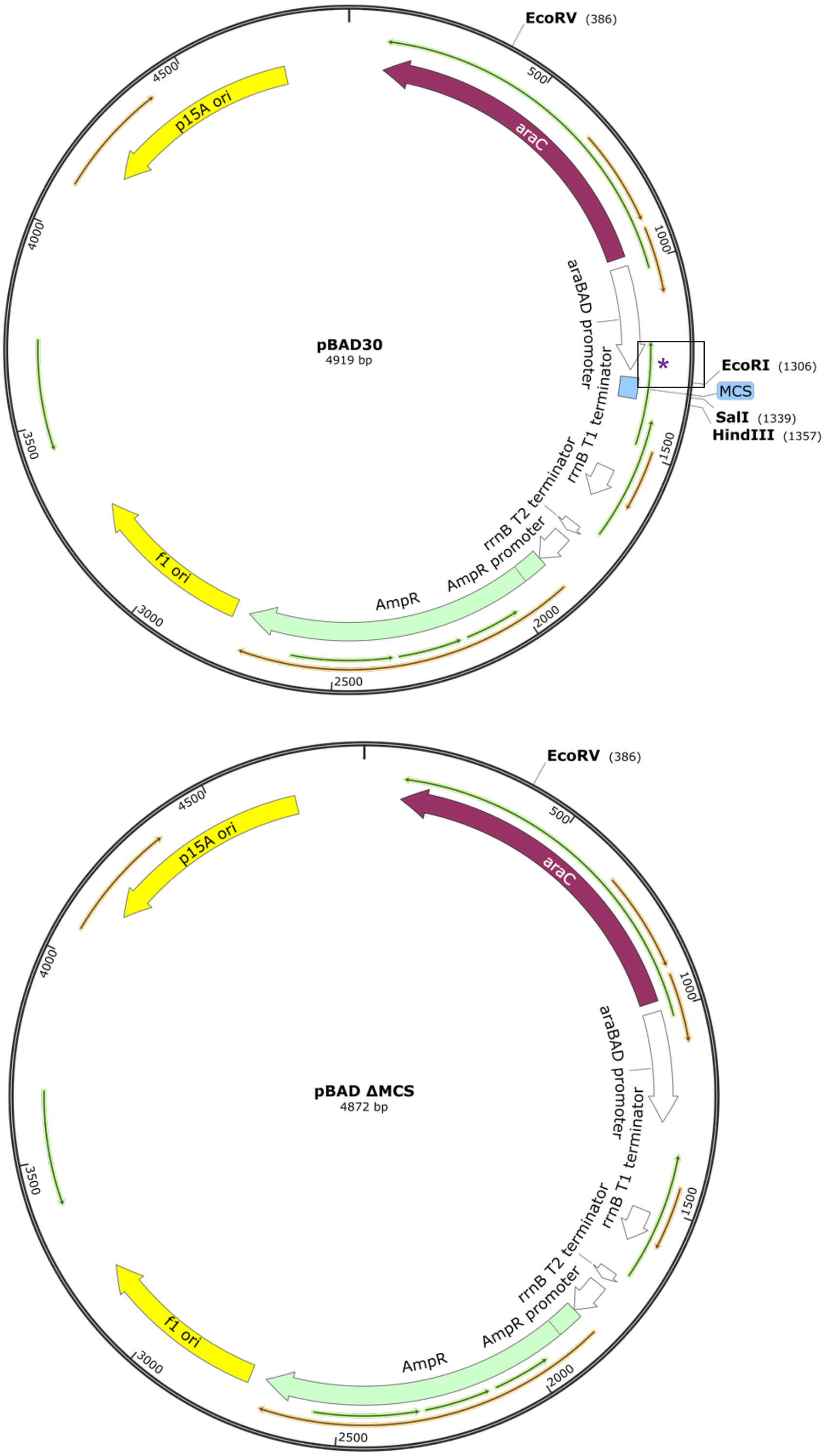

*Predicted ORF:
MGSGGTTALLPPGKFCFIRPLLRSDLICIRLKIFSHPPKQPSLHACRSTLEDPRVPSSNSLAQKNGYGETVESCDKKRQVGSANLMDKNAMA*

Figure S4. Plasmid maps of pBAD30 and pBAD30 ΔMCS. Each map shows restriction sites for the enzymes used in the digestion analysis (Figure S3). Orange and green arrows represent predicted open reading frames > 50 amino acids with an ATG start codon. A hypothetical 92 amino acid ORF present in pBAD30 but not the ΔMCS construct is indicated by a purple asterisk (*) and its sequence shown at the bottom of the figure.

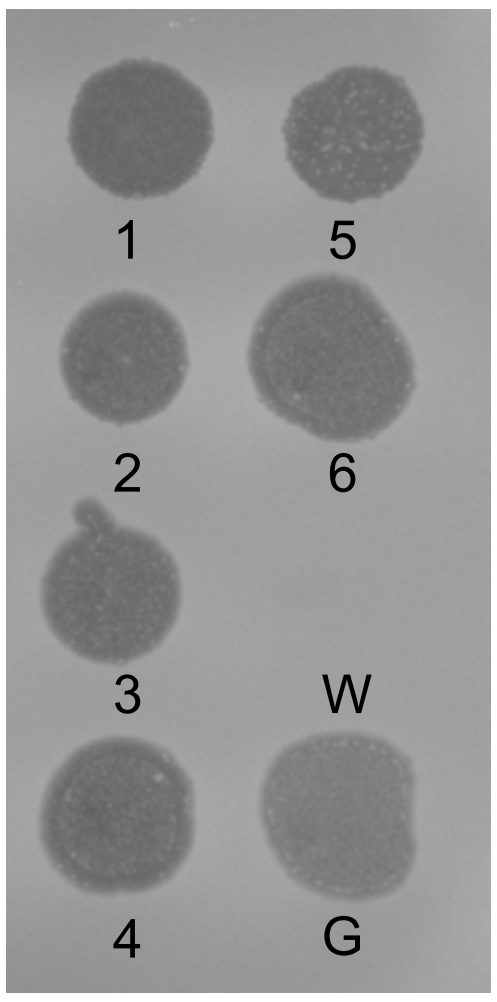

Figure S5. Screening for W3110 lysogens. Six candidate W3110 lysogens (labelled 1 to 6) were grown in LB (37 °C, 200 rpm) and 100 μL of culture removed after 180 min growth. After centrifugation, 4 μL of supernatant was added to top agar containing W3110. Supernatants from W3110 (labelled W) and GR536 (G) were used as negative and positive controls for ϕ80 production, respectively.

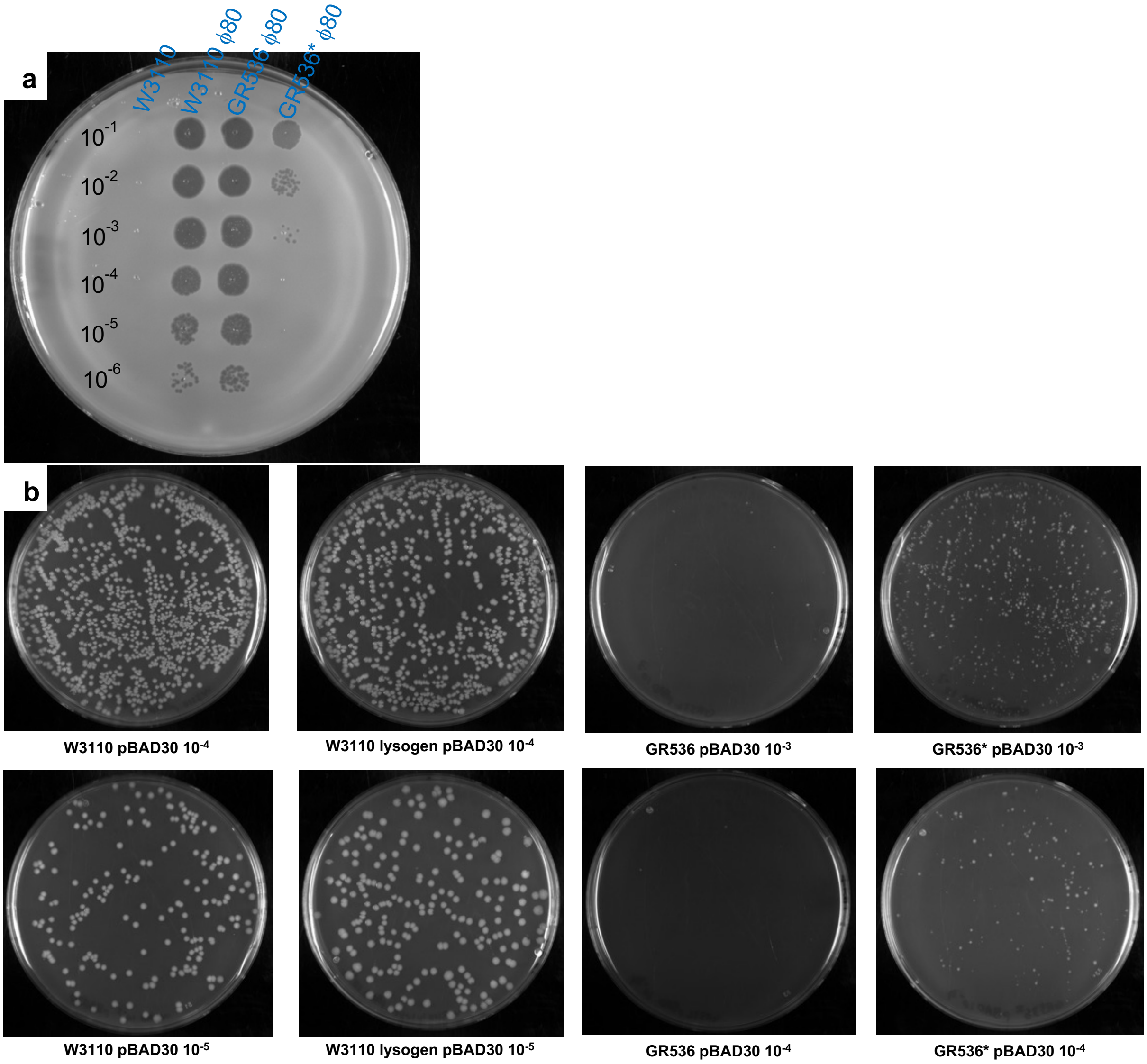

Supplementary Figure S6. Phage production by different strains containing pBAD30 results in different amounts of cell lysis. All strains were grown in IRM + 100 μg/mL ampicillin (37 °C, 200 rpm) and 100 μL of culture removed at OD_{600} values as follows: W3110, 0.146, ~100 min; W3110 ϕ80 lysogen, 0.136, ~100 min; GR536 ϕ80, 0.126, ~160 min, GR536* ϕ80, 0.160, ~160 min). Panel a, ϕ80 titre determined by 10-fold serial dilutions and spotting 4 μL onto top agar containing W3110 (37 °C, 14 h). Panel b, colony forming units determined from serial dilution of a separate aliquot and plated on LB agar without antibiotic (37 °C, 14 h)

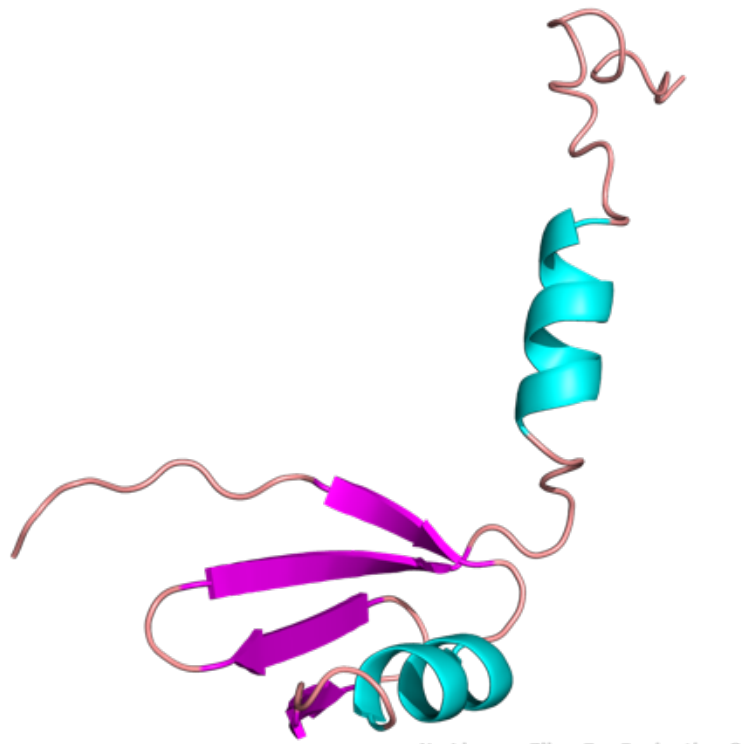

MTDQATPNLPSRDFDSTAAFYERLGFGIVFRDAGWMILQRGDLMLEFFAHPSFKSALKFL YFLENRNFGIGTKEDIHMDHG*AILTCAAKAENERPASGSRLRRMVLTKGVVLRKTRNGVIPS PSSFLLCSAMSRTRRVKRTIEIRIIFLTVVKCLSSTA*

Figure S7. **Identification of a hypothetical 159 amino acid ORF fusion (nucleotide position 757305 to 757784) resulting from the *kan* cassette disruption of *fecABCDE*.** The italicised residues result from the in-frame sequence of *fecA*. The amino acid residues in the AF predicted structure (ID: A0A8A8S825) are highlighted in bold. The predicted structure is annotated as a homologous to a bleomycin resistance protein.
